# Intracerebral Transplantation of Neural Stem Cells Restores Manganese-Induced Cognitive Deficits in Mice

**DOI:** 10.14336/AD.2020.0717

**Published:** 2021-04-01

**Authors:** Huijuan Shu, Zhongxin Guo, Xiangren Chen, Shuya Qi, Xinxin Xiong, Shuang Xia, Qingyun Huang, Ling Lan, Jiangu Gong, Shaoming Huang, Boning Yang, Guohe Tan

**Affiliations:** ^1^Key Laboratory of Longevity and Aging-related Diseases of Chinese Ministry of Education, Guangxi Collaborative Innovation Center for Biomedicine & Guangxi Key Laboratory of Regenerative Medicine, Center for Translational Medicine, Guangxi Medical University, Nanning, Guangxi, China.; ^2^Department of Human Anatomy, School of Basic Medical Sciences, Guangxi Medical University, Nanning, Guangxi, China.; ^3^China-ASEAN Research Center for Innovation and Development in Brain Science, Nanning, Guangxi, China.

**Keywords:** neural stem cells, cell transplantation, manganese neurotoxicity, learning and memory

## Abstract

Manganese (Mn) is a potent neurotoxin known to cause long-lasting structural damage and progressive cognitive deficits in the brain. However, new therapeutic approaches are urgently needed since current treatments only target symptoms of Mn exposure. Recent studies have suggested a potential role for multipotent neural stem cells (NSCs) in the etiology of Mn-induced cognitive deficits. In this study, we evaluated the effect of direct intracerebral transplantation of NSCs on cognitive function of mice chronically exposed to MnCl_2_, and further explored the distribution of transplanted NSCs in brain tissues. NSCs were isolated and bilaterally injected into the hippocampal regions or lateral ventricles of Mn-exposed mice. The results showed that many transplanted cells migrated far away from the injection sites and survived *in vivo* in the Mn-exposed mouse brain, implying enhanced neurogenesis in the host brain. We found that NSCs transplanted into either the hippocampal regions or the lateral ventricles significantly improved spatial learning and memory function of the Mn-exposed mice in the Morris water maze. Immunofluorescence analyses indicated that some surviving NSCs differentiated into neurons or glial cells, which may have become functionally integrated into the impaired local circuits, providing a possible cellular basis for the improvement of cognitive function in NSC-transplanted mice. Taken together, our findings confirm the Mn-induced impairment of neurogenesis in the brain and underscore the potential of treating Mn exposure by NSC transplantation, providing a practical therapeutic strategy against this type of neurotoxicity.

Despite its essentiality, manganese (Mn) is one of the most common environmental contaminants and is a potent neurotoxin that can induce toxicity in humans [1]. The excessive accumulation of Mn in the brain can cause a variety of neurochemical changes that could result in permanent structural damage, even at low exposure levels [1]. Thus, Mn exposure from various sources induces a neurodegenerative condition known as manganism that is associated with distinct neurological symptoms [2], including cognitive and neurobehavioral deficits that have been described in humans [3, 4]. Previous studies have also shown similar neurological phenotypes following elevated Mn exposure in adult and early postweaning rodents, as well as in non-human primates [5, 6]. Particularly, children are highly vulnerable to Mn toxicity [7]. However, current therapeutic approaches are only palliative and do not compensate for the progressive structural damage and neuronal loss induced by an excessive accumulation of Mn in the brain [8]. Thus, Mn exposure remains a public health problem due to its extensive use in common household products like batteries, paints, and gasoline [9].

In the mammalian brain, the hippocampus plays an essential role in learning and memory functions, including spatial memory [10-12]. A recent study using synchrotron X-ray fluorescence (XRF) microscopy revealed that the hippocampal dentate gyrus (DG) and CA3 area were the regions with the greatest accumulation of Mn in Mn-exposed rats [13]. and several recent reports have identified the hippocampus as one of the key brain areas affected by Mn toxicity [14], where the metal has been found to selectively accumulate. Also, deficits in hippocampus-dependent spatial cognition often occur following the excessive accumulation of Mn in the brain [15]. New cells are generated continuously throughout postnatal life in distinct functional regions of the mammalian brain [16, 17], including the subgranular zone (SGZ) within the hippocampus, as well as the subventricular zone (SVZ), through a process called neurogenesis. Mounting evidence supports a strong link between adult neurogenesis and spatial learning and memory, whereas impaired neurogenesis is often associated with a number of neurodegenerative diseases [18, 19]. We and others have previously detected a decrease in the proliferation of multipotent neural stem cells (NSCs) within the hippocampal SGZ that was suggestive of disrupted neurogenesis that closely coincided with deficits in spatial recognition. Consistently, studies from other laboratories have also found that NSCs are the prime targets of Mn toxicity in both the DG and the SVZ [20]. These NSCs are stem cell-like precursors that exist, predominantly, in these two brain regions to produce somatic cells such as neurons and glial cells in response to a variety of environmental conditions [20]. Furthermore, converging evidence has shown that NSCs in the brain undergoing differentiation and maturation play important roles in learning and memory functions [19, 21], suggesting the potential of NSC transplantation as a promising tool to compensate for neuronal loss and structural damage in the adult hippocampus [20]. This would provide an indispensable source of new cells to support neuroplasticity and granule cell turnover that could lead to recovery from cognitive impairments [22, 23]. Actually, a series of studies to date have established the therapeutic efficacy of exogenous NSC transplantation in treating neurodegenerative disorders and central nervous system (CNS) injuries in both humans and experimental animals, including Parkinson's disease and Alzheimer's disease, as well as in promoting recovery from chronic lead exposure [24-27]. Based on the observation that Mn causes NSC impairment, this current study, therefore, aimed to evaluate whether intracerebral NSC grafts could restore Mn-induced cognitive deficits in the rodent brain. In this work, we present experimental data showing that injection of *in vitro* cultured NSCs into the mouse brain, both intraventricularly and intrahippocampally, can improve the spatial cognitive function of Mn-exposed mice that is associated with cellular differentiation and integration into the local parenchyma of transplanted cells. The results described here provide a rationale for further investigation of NSCs for the treatment of heavy metal-induced toxic encephalopathies associated with impaired neurogenesis and neurobehavioral deficits.

## MATERIALS AND METHODS

### Animals and treatment

Male Kunming or C57/BL6 mice were obtained from the animal breeding colony in the Animal Centre of Guangxi Medical University, China. Nestin-green fluorescent protein (GFP) (Stock No.033927) and CAG-eGFP mice (Stock No.006567) were obtained from Jackson Laboratory (United States), and have been described previously [28, 29]. All mice were housed in temperature-controlled rooms, under a 12h light/12h dark cycle, and given food and water *ad libitum*. All the studies were approved by the Institutional Animal Care and Use Committee of Guangxi Medical University and were performed in compliance with the U.S. National Institutes of Health Guide for the Care and Use of Laboratory Animals. Mice were randomly assigned to one of several groups (seven mice in each group) receiving either manganese chloride or vehicle. MnCl_2_·4H_2_O dissolved in sterile saline was administrated to mice by intraperitoneal injection at a volume of 1 mL/kg body weight at a dose of 5mg/mL (referred to as low-dose exposure), 20 mg/mL (referred to as middle-dose exposure), and 50 mg/mL (referred to as high-dose exposure), once daily for two consecutive weeks. We and others have previously demonstrated that daily intraperitoneal administration of MnCl_2_ at doses exceeding 5mg/kg body weight could produce a significantly elevated Mn concentration in the hippocampus in a dose-dependent manner after 14 consecutive days [20, 30]. A daily equivalent volume of sterile saline was given to the sham control animals. Transplantation of NSCs was performed on high-dose Mn-exposed mice and their respective parallel controls.

### Culture, labeling and transplantation of NSCs

Mn-exposed mice received bilateral intrahippocampal or intracerebral NSC transplantation. Appropriate sham controls were run in parallel. Transplanted NSCs were harvested from postnatal day 0 mice, grown as suspended neurospheres, and transplanted at passage 7. In detail, the embryos were removed from the horns of deeply anesthetized (5% chloral hydrate, 10g/0.1mL) pregnant mice and transferred to a sterile dish on ice for dissection. After decapitation, the brain was dissected and transferred to a sterile Petri dish containing 10~20 mL of Hanks’ Balanced Salt Solution (HBSS). The neural tissue was incubated in 0.1% trypsin at 37 °C for 20 min, then washed once with the medium. A homogenous suspension was made by repeated trituration with a fire-polished Pasteur pipette, then subsequently processed to culture at 37 °C. Primary NSCs were subcultured every seven days. Before transplantation, neurospheres were collected, trypsinized, washed, triturated, and filtered through a 70-μm mesh. NSCs were then counted and resuspended at 100,000 cells/μL in vehicle (1× HBSS with 20 ng/mL human epidermal growth factor (hEGF)).

Bilateral stereotactic deliveries of NSCs or vehicle were performed using a stereotaxic apparatus; the coordinates relative to Bregma are indicated as follows. For intrahippocampal implantation, injections were targeted to the hippocampal fissure, a cell-sparse region located just dorsal to the molecular layer of the DG (AP, -1.0 mm; ML, -0.3 mm; DV, -2.0 mm). For intraventricular implantation, injections were targeted to the lateral ventricles (AP, +1.5 mm; ML, -2.0 mm; DV, -2.0 mm). Host mice were anesthetized with sodium pentabarbitone (40 mg/kg body weight), placed in the stereotaxic apparatus, and the coordinates for implantation into the hippocampus or lateral ventricles were accurately determined. Each animal received an intrahippocampal or intraventricluar injection of either 2 μL of the NSC suspension per side or vehicle (1×HBSS) as a control treatment, using a 5 μL Hamilton microsyringe (33-gauge) at an injection rate of 0.5 μL/min. Following injections, the incisions were sutured, and all mice received an intramuscular injection of 100 kU of penicillin to prevent infection after the stereotaxic operation. Mice were assessed behaviorally, biochemically, and morphologically two months after the stereotaxic transplantation of NSCs.

### Open field test

The open field was used to measure spontaneous locomotor activity in the full arena. Defined center and perimeter zones were assessed using activity chambers in connection with an automated recording and video tracking system (Ethovision XT software). Spontaneous locomotor activity tests were performed at two and four weeks after transplantation. Mice were placed individually in the center of a 40 cm × 40 cm × 40 cm transparent plastic chamber in a darkened room and were allowed to explore freely for 30 min; movement was tracked with a digital video camera under infrared light. Between subjects, the arena was swabbed with 70% ethanol solution to prevent possible interference from animal odors.

### Morris water maze

The Morris water maze (MWM) was performed as described previously [31] to assess changes in spatial memory performance. Animals were brought to the behavior room and habituated to a circular black pool (120 cm diameter) filled with opaque water maintained at 25 °C. During training, mice were paced in the pool and allowed to find and climb onto a submerged platform (11 cm diameter) located 1 cm below the water surface at a fixed point of quadrant three; four trials were performed per day. After five consecutive days of training, the escape platform was removed, and mice were tested 24 h later to assess retention of spatial memory in a probe trial. For the probe trial, the latency to reach the submerged platform was measured and the number of times the mouse crossed the platform location was recorded. Swim paths were monitored using an automated tracking system (Ethovision XT software).

### Immunostaining

The mice were anesthetized (5% chloral hydrate, 10g/0.1mL) and transcardially perfused with 4% paraformaldehyde (PFA) in 0.1mol/L PBS. For light-microscopic immunehistochemistry (IHC), 40 μm-thick brain cryosections were then prepared using a cryostat (Leica CM1900, Hessen, Germany). Free-floating brain sections were incubated in 3% (vol/vol) H_2_O_2_ for 10 min to block endogenous peroxidase activity, then in blocking buffer (5% (wt/vol) bovine serum albumin (BSA), 10% (vol/vol) normal goat serum (NGS), 0.25% (vol/vol) Triton X-100) for 1 h to block non-specific background staining. Sections were then incubated for 48 hours with one of the following primary antibodies: mouse anti-nestin (1:300; Chemicon; MAB5326) for neural progenitor cells or rabbit anti-glial fibrillary acidic protein (GFAP) (1:500; Millipore; MAB360) for astrocytes. Afterward, we processed these sections for free-floating IHC and finally 3,3',5,5'-tetramethylbenzidine (TMB, Amersham) staining for visualization. Sections were then washed with PBS, air-dried on gelatin-coated slides, dehydrated with graded ethanol concentrations, and finally cleared with xylene and mounted with dibutylphthalate polystyrene xylene (DPX) mounting medium (Fluka; Sigma). Sections were observed under bright field microscopy using a Leica DMi8 microscope. Immunoreactive cells were counted using Image Pro-Plus 6.0 software, and the number of cells in each section was divided by the size of the respective sample area.

**Figure 1.**
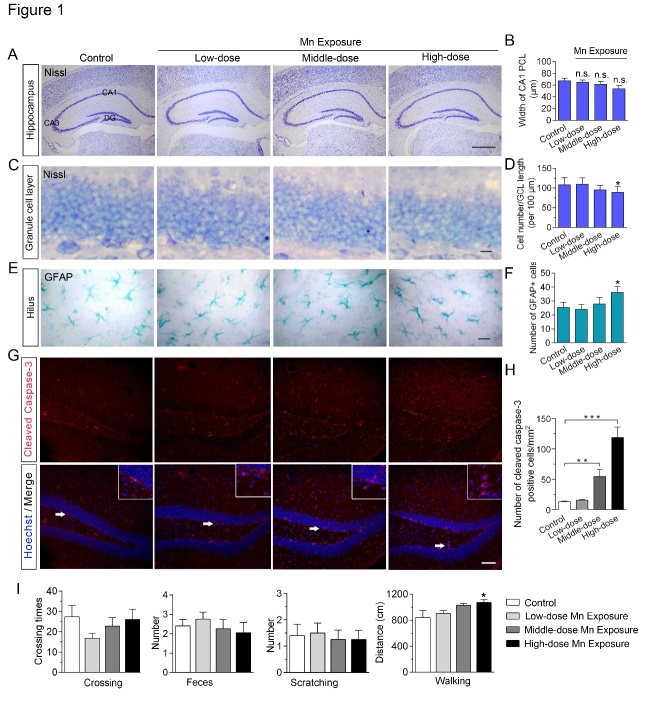
Effects of subchronic Mn exposure on mouse hippocampal morphology and locomotor behavior in the open field test. (A) Nissl staining of coronal brain sections reveals no differences in gross morphology of the hippocampus between Mn-exposure mice and their controls. DG, dentate gyrus. Scale bar, 300 μm. (B) Quantitative analysis of neuronal density in mouse hippocampal CA1 area after Mn exposure. PCL, pyramidal cell layer. (C) Representative Nissl staining of the granule cell layer (GCL) in the hippocampal DG. Scale bar, 15 μm. (D) Statistical analysis of cell numbers in the GCL of control and Mn-exposed mice. (E) Representative immunohistochemistry images of astrocytic glial fibrillary acidic protein (GFAP) immunoreactivity in the hilus of the mouse hippocampus. Scale bar, 15 μm. (F) Quantification of the number of astrocytes in control and Mn-exposed mice. (G) Immunofluorescent labeling of cleaved caspase-3 in brains of control and Mn-exposed mice. Hoechst staining was used to label the DNA in the cell nuclei. Magnification of the areas pointed to by the arrows is shown in the upper right corner of each panel. Scale bar, 60 μm. (H) Quantitative analysis of activated caspase-3-positive cells in mouse hippocampal area after Mn exposure. *n* = 4-6 in each group. (I) The histograms show quantitative analysis of locomotor activity of Mn-treated mice and their controls in the open field test. *n* = 7 in each group. Error bars represent means ± SEM. *P < 0.05, ** *P* < 0.01, *** *P* < 0.01 vs control; data are analyzed by one-way ANOVAs with *post hoc* Dunnett’s tests.

For immunofluorescent labeling, mouse brain sections were washed with PBS, then incubated in blocking buffer for 1 h at room temperature (RT), followed by overnight incubation with primary antibodies at 4 °C (rabbit anti-cleaved caspase-3 (1:500; Cell Signaling Technology; #9664) for apoptotic cells, goat anti-doublecortin (DCX) (1:1,000; Santa Cruz; sc-8066) for immature neurons, mouse anti-NeuN (1:1,000; Millipore; MAB377) for mature neurons, or rat anti-BrdU (1:200; Abcam; ab6326) to assess the proliferation of NSCs). The sections were then washed with PBS and incubated with Alexa Fluor-conjugated secondary antibodies for 2 h at RT (1:1,000). Hoechst 33342 (Beyotime, 1:5,000) was used for counterstaining of the cell nuclei. All sections were mounted using Fluoromount-G (SouthernBiotech).

For BrdU immunostaining of cultured NSCs, cells were incubated with 20 μM BrdU for 6 h; they were then washed and subsequently fixed with 4% PFA for 25 min, followed by treatment with 1N HCl for 25 min to denature the chromosomes within the cells. The cells were then processed according to the standard procedures [27].

### Western blotting

DG tissue was isolated from the hippocampal formation and the total protein was extracted using a standard assay for western blotting [32]. Lysed protein samples were resolved on 8-15 % precast SDS-polyacrylamide gels under electrophoresis (BioRad, Richmond, CA), transferred to nitrocellulose/polyvinylidene difluoride membranes (Millipore, Bedford, MA), then subjected to immunoblotting using primary antibodies against rabbit anti-tropomyosin receptor kinase B (TrkB) (1:500; Santa Cruz; sc-20542), mouse anti-brain-derived neurotrophic factor (BDNF) (1:200; LSBio; LS-C196746), and mouse anti-synaptophysin (SYP) (1:1,000; SYSY; Cat. No. 102011). An antibody against β-actin (1:2,000; Abcam) was used to control for protein loading. Protein bands were visualized by enhanced chemiluminescence with an ECL Plus chemiluminescence detection kit (Amersham). Densitometric analysis of the bands was performed using a Quantity One system (version 4.6, Bio-Rad).

### Nissl staining

For evaluation of gross histological changes, brain slices were stained with 0.1% cresyl violet (Sigma, USA) according to the protocol described previously [33]. Viable neurons were defined as those in which a clear nucleus could be seen. Images were captured under a Leica DMi8 inverted microscope. Nissl-positive cells were counted automatically using Image Pro-Plus 6.0 software (Media Cybernetics, USA), and the number of cells in each section was divided by the length of the respective cell layer sampled.

### Statistical Analysis

For all statistical analyses, investigators were unaware of treatment groups. All statistical analyses were performed using IBM Statistical Package for the Social Sciences 26.0 (SPSS 26.0) software. Comparisons between multiple groups were performed by analysis of variance (ANOVA) followed by *post hoc* Dunnett’s tests. Comparisons between two individual groups were performed using paired or unpaired students’ *t*-tests. Data are presented as means ± SEMs. Differences were considered statistically significant at *P* values < 0.05.

## RESULTS

### Mn exposure impairs neurogenesis in the mouse brain

As a common neurotoxic metal, excessive accumulation of Mn is expected to cause a series of morphological and cellular changes within the mammalian brain [4]. Therefore, we investigated the structure and morphology of Mn-exposed mouse brains, and examined behavioral phenotypes using the open field test and the MWM. Nissl staining revealed no significant changes in gross morphological structure or neuronal distribution patterns in the brains of mice following Mn exposure at the dosages we used (Fig. 1A-D). Quantitative analysis revealed normal neuronal density in the hippocampal CA1 area (Fig. 1A and B) but decreased neuronal numbers in the granule cell layer of the DG (Fig. 1C and D), an area key for spatial learning and memory [21], after high-dose Mn exposure. Moreover, the IHC results showed increased numbers of GFAP-immunoreactive astrocytes in the hippocampus of Mn-exposed mice (Fig. 1E and F), reflecting glial cell activation in response to Mn exposure [13]. Moreover, we assessed the cells with caspase activation (Fig. 1G and H) to evaluate the extent of cellular apoptosis induced by Mn exposure in the hippocampus. Immunofluorescence showed the numbers of cleaved caspase-3-positive cells in the middle-dose group (54.86 ± 11.33; *P* = 0.003, *vs* control) and high-dose group (118.9 ± 16.79; *P* = 0.0001, *vs* control) were markedly increased compared with the control group (13.19 ± 1.34), implying a loss of cells in the hippocampus within the Mn-exposed brains. In addition, a 50 mg/kg dose of daily Mn exposure caused a slight increase in the total distance traveled and more time spent in the center zone of the arena compared to control animals (*P* < 0.05) (Fig. 1I) in the open field test, consistent with the hyperlocomotion induced by Mn that has been reported in other neonatal animal studies [34].

**Figure 2.**
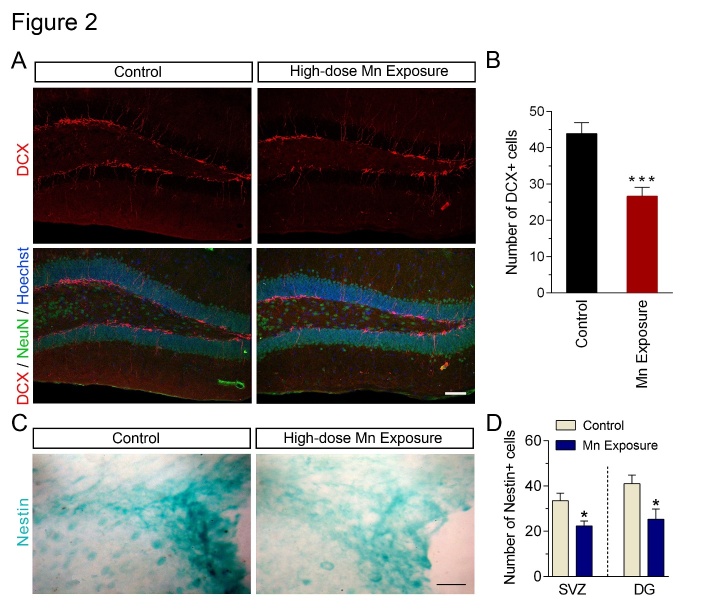
Manganese (Mn) exposure impairs the number of newly generated neurons in the mouse brain. (A) Representative images showing decreased numbers of newly generated double-cortin (DCX)-immunopositive neurons (red) in the dentate gyrus of the Mn-exposed mouse brain. NeuN is used as a marker of mature neurons (green) and Hoechst staining is used to label DNA in the cell nuclei (blue). Scale bar, 100 μm. (B) Quantification of the number of DCX-expressing cells in the hippocampal dentate gyrus of control and high-dose Mn-exposure groups. *n* = 11 for control group; *n* = 8 for Mn-exposure group. Error bars represent means ± SEM. ***P < 0.001, *vs* control; data are analyzed by unpaired *t*-test. (C) Representative images showing nestin-immunopositive neural progenitor cells in the lateral ventricle of control an Mn-exposed mouse. Scale bar, 30 μm. (D) Quantification of the number of nestin-positive cells in the subventricular zone (SVZ) and dentate gyrus (DG) in control and high-dose Mn-exposure groups. *n* = 7 for each group. Error bars represent means ± SEM. * *P* < 0.05, *vs* control; data are analyzed by unpaired *t*-test.

To further confirm the extent to which neuronal differentiation from NSCs in the DG was affected by Mn exposure, we quantified the numbers of newly generated cell populations expressing DCX in the DG after Mn exposure (Fig. 2A). As shown in Figure 2, the numbers of DCX-positive cells in the high-dose Mn exposure group (26.63 ± 2.49; *P* = 0.0005, *vs* control) were significantly reduced relative to the control group (43.91 ± 2.93) (Fig. 2B). Moreover, we found that the number of nestin-positive neural progenitor cells was markedly decreased in both the SVZ and the DG of Mn-exposed mouse brains (Fig. 2C and D). Together, these results further support the idea that adult neurogenesis is highly susceptible to the deleterious effects of Mn toxicity in the brain.

### Characterization, transplantation, and in vivo survival of NSCs

**Figure 3.**
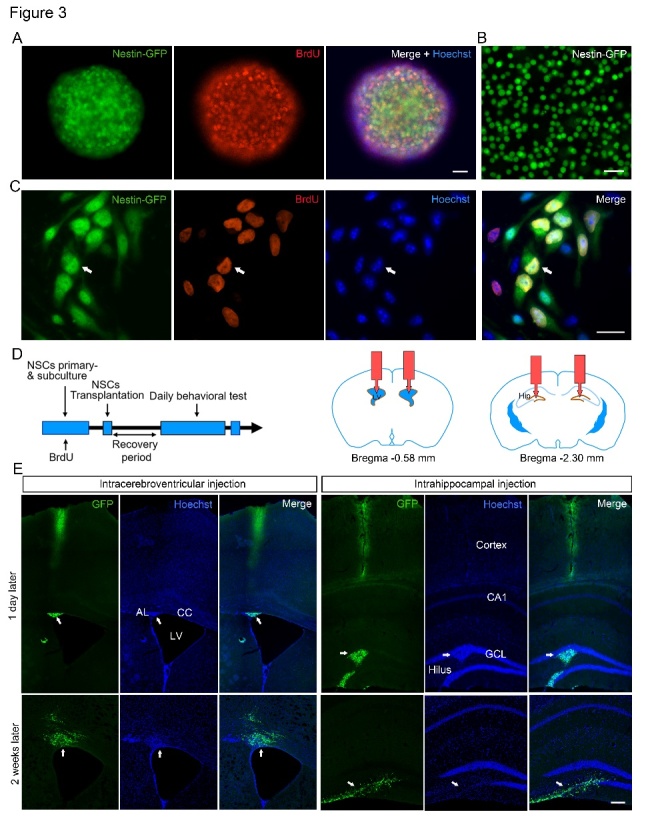
Characterization and transplantation of cultured neural stem cells (NSCs). (A) Immunofluorescent labeling showing the proliferation of neurospheres with transgenic nestin-green fluorescent protein (GFP) (green) and BrdU (red) immunoreactivity, and Hoechst staining of DNA in cell nuclei (blue). (B) Single cell suspension of cultured nestin-GFP neurospheres before transplantation. (C) Immunofluorescent labeling of the cultured NSCs showing their ability to self-renew and differentiate after attachment *in vitro*. Scale bar, 50 μm. (D) Schematic depiction of the timeline of NSC culture, transplantation, recovery, and behavioral testing (left), and the coordinates of the intracerebroventricular and intrahippocampal NSC injection sites (right). (E) Photomicrographs showing eGFP-positive NSC transplants in the lateral ventricle (left panels) or hippocampal dentate gyrus (right panels) of Mn-exposed mice 1 day (upper panels) and 2 weeks (lower panels) after intracerebral transplantation, respectively. Many surviving engrafted eGFP-positive NSCs are present at the injection site and the adjacent regions. Note that many engrafted NSCs remain mostly undifferentiated and clustered *in vivo* after 1-day post-transplantation. AL, angulus

Based on the strong link between adult hippocampal neurogenesis and learning and memory functions [20], a Mn exposure-induced reduction of hippocampal neurogenesis might cause deficits in spatial learning and memory tasks to a certain extent. Thus, we evaluated behavioral changes after enhancing neurogenesis by transplantation of cultured NSCs in the Mn-exposure model. Firstly, we isolated NSCs from nestin-GFP transgenic mice at postnatal day 0, as previously described. In our experiments, approximately 100,000 GFP-positive NSCs were obtained from a batch of cultured neurospheres. After five days *in vitro*, cells formed large spherical colonies and a large number of them exhibited BrdU immuno-positive labeling (Fig. 3A), indicating high proliferative activity of the NSCs. Single cell suspensions of cultured GFP-positive neurospheres are multipotent *in vitro* before transplantation (Fig. 3B), and these cultured NSCs exhibited the ability to self-renew and differentiate after cell attachment *in vitro* (Fig. 3C). Subsequently, we transplanted eGFP-labeled transgenic NSCs bilaterally into the lateral ventricles or the hippocampal DG of Mn-exposed mice (Fig. 3D). In an initial pilot experiment, mice were sacrificed one day and two weeks after transplantation, and the transplanted cells were located in brain sections. Although 10,000 cells were transplanted at one site, only 20-30% of the cells survived the first two weeks (Fig. 3E). As shown in Figure 3E, numerous eGFP-positive cells could be identified in clusters along the needle tracts within the brain of the Mn-exposed mice receiving intracerebroventricular administration of NSCs; similarly, the largest number of eGFP-positive cells was observed in the DG of the mouse hippocampus, close to the injection site, after intrahippocampal transplantation. A significant number of transplanted cells had also migrated from the injection site within several weeks, either laterally along the lateral ventricles (Fig. 3E, left panel) or in the hilus and DG region of the mouse hippocampus (Fig. 3E, right panel), brain regions important for ongoing neurogenesis throughout postnatal life [17].

In the transplanted animals, eGFP-positive cells were observed in multiple brain areas beyond the injection sites, as well, including the cortex above the sites, the angulus lateralis and the ependymal lining of the lateral ventricles, and the corpus callosum. Importantly, we observed that the NSCs remained mostly in the center of the injection site one day post-transplantation (Fig. 3E, upper panel), and a large number of differentiated NSCs were seen under confocal microscopy after eGFP-NSC transplantation at two weeks (Fig. 3E, lower panel), implying their functional involvement in processes in the host brain. It is important to note that two weeks after transplantation, sections that were further away from the injection site contained numerous surviving and differentiated eGFP-positive NSCs. These observations were similar to those reported previously [35], indicating that engrafted NSCs enhanced neurogenesis in the host brain following NSC transplantation.

**Figure 4.**
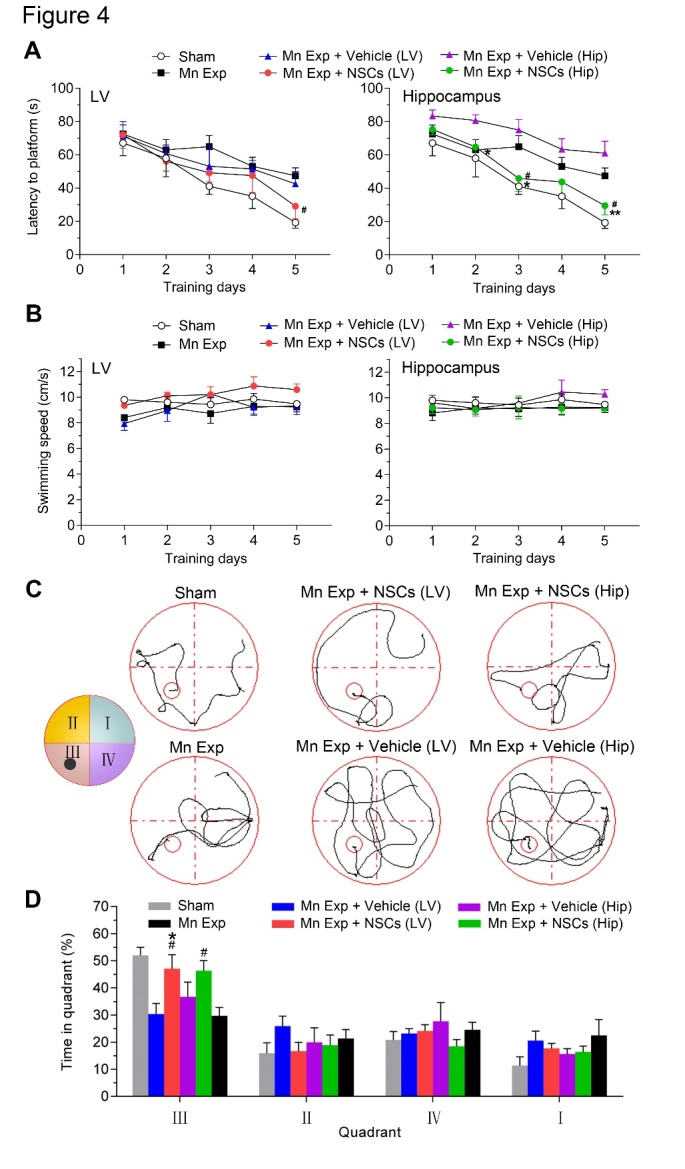
Neural stem cell (NSC) transplantation ameliorates learning impairments in Mn-exposed mice. (A) The time to reach the platform (latency) for spatial navigation of mice in the Morris Water Maze (MWM). Left panel, transplantation of NSCs into the lateral ventricle (LV); right panel, transplantation of NSCs into the hippocampus (Hip). The mean escape latency was given for different test days. (B) Analysis of swimming speed of each group of mice across training days in the MWM test. (C) Representative search paths of mice on day 5 of testing in the MWM. A schematic depiction of the quadrants of the MWM test is shown on the left. The black dot represents the location of the platform. (D) The mean percentage of time spent by each group in each quadrant of the MWM in the probe trial on day 5. III is the target quadrant. *n* = 7 for each group. Error bars represent means ± SEM. * *P* <0.05 and ** *P* <0.01, *vs* Vehicle; ^#^*P* <0.05, *vs* Mn-exposure mice; data are analyzed by two-way ANOVAs.

**Figure 5.**
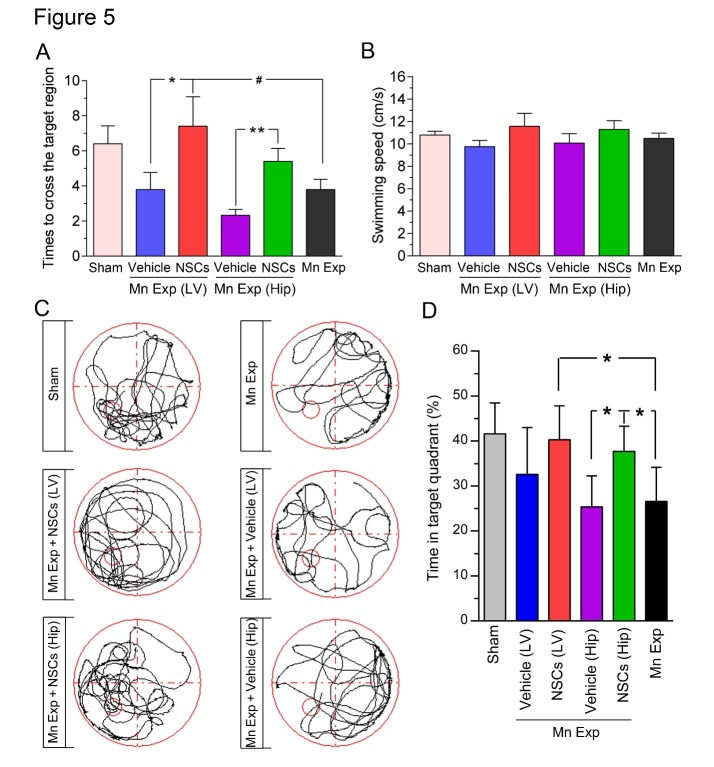
Neural stem cell (NSC) transplantation rescued the memory deficits observed in Mn-exposed mice in the Morris water maze (MWM). The frequency to pass the target position (A) and the swimming velocity (B) in the probe trial of the MWM on day 6. *n* = 7-10 for each group. Error bars represent means ± SEM. * *P*<0.05, ** *P*<0.01, *vs* Vehicle; ^#^ *P*<0.05, *vs* Mn-exposure mice. (C) Representative search paths for mice of each group in the probe trial. (D) Quantitative analysis of mean percentage of time spent in the target quadrant (III). * *P*<0.05; data are analyzed by one-way ANOVA with *post hoc* Bonferroni’s test.

### NSC transplantation improves spatial cognitive behavior of Mn-exposed mice

Increasing evidence from both clinical investigations and experimental animal models suggests that excessive deposition of Mn in the hippocampus causes neurobehavioral alterations such as impaired spatial learning, memory loss, and cognitive deficits in rodents, non-human primates, and humans [6, 34], that is closely associated with impaired neurogenesis in the brain [20]. To explore the potential therapeutic effects of NSC transplantation to treat toxicity resulting from Mn exposure, we performed bilateral intracerebral injections of cultured NSCs into the lateral ventricles or hippocampus of high-dose Mn-exposed mice (Fig. 3) and measured subsequent behavioral performance following the transplantation. No mortality and no obvious body weight losses were observed in Mn-exposed mice after the transplantation. Transplantation did not significantly affect spontaneous locomotor activity in either NSC-transplanted or vehicle-transplanted mice (data not shown).

We then evaluated the spatial cognition of NSC-transplanted mice using the MWM test (Fig. 4A-D), a common behavioral task used to assess changes in spatial learning and reference memory performance. Spatial cognitive deficits in the MWM as a result of Mn exposure were observed in sham-operated Mn-exposed mice compared with the sham-operated saline-treated mice, consistent with a previous report [36] (Fig. 4A and B). NSC transplantation did not affect the swimming velocity of NSC-transplanted mice in the MWM (Fig. 4B); however, Mn-exposed mice that received intraventricular NSC transplants displayed improved spatial learning on day 5, as evidenced by the significantly decreased latency to reach the platform (Fig. 4A, C and D). Intriguingly, Mn-exposed mice that received intrahippocampal NSC transplants also required less time than the control mice to locate the hidden platform consistently from the second day of training (*P* = 0.045 for day 3, *P* = 0.0087 for day 5, *versus* vehicle-transplanted) (Fig. 4A), reflecting robust restoration of spatial learning ability in the Mn-exposed mice. These findings indicate that both intra-cerebroventricular and intrahippocampal NSC transplantation alleviated the impairment of spatial learning in Mn-exposed mice.

To further assess the retention of spatial memory in NSC-transplanted mice, we evaluated their performance in the probe trails conducted thereafter (Fig. 5A-D). On the 6^th^ day of training in the MWM, normal control (sham) mice and Mn-exposed mice required 6.40 ± 1.03 and 3.79 ± 0.58, respectively, to cross the target region in the probe trial; meanwhile, the times required for the intraventricular or intrahippocampal vehicle-transplanted mice to cross the target region were 3.86 ± 0.97 and 2.33 ± 0.34, respectively. In contrast, at the same timepoint, the intraventricular NSC-transplanted mice required 7.40 ± 1.69 (*P* = 0.032 *versus* vehicle) and the intrahippocampal NSC-transplanted mice required 5.41 ± 0.75 (*P* =0.024 *versus* vehicle) (Fig. 5 A and C). Meanwhile, the time that the NSC-transplanted mice spent in the target quadrant exceeded those of the vehicle-treated or control Mn-exposed mice (*P* < 0.05), both intraventricularly and intrahippocampally, indicating improved memory retention following NSC transplantation (Fig. 5D). No significant effects of experimental treatments or interactions among the independent variables were revealed by an ANOVA for random swimming speed in the probe trail (*F* = 0.833, *P* = 0.540) (Fig. 5B). Taken together, the above results suggest that exogenous NSC transplantation can efficaciously restore the spatial memory retention in Mn-exposed mice.

### Migration and differentiation of transplanted NSCs in the host brain

**Figure 6.**
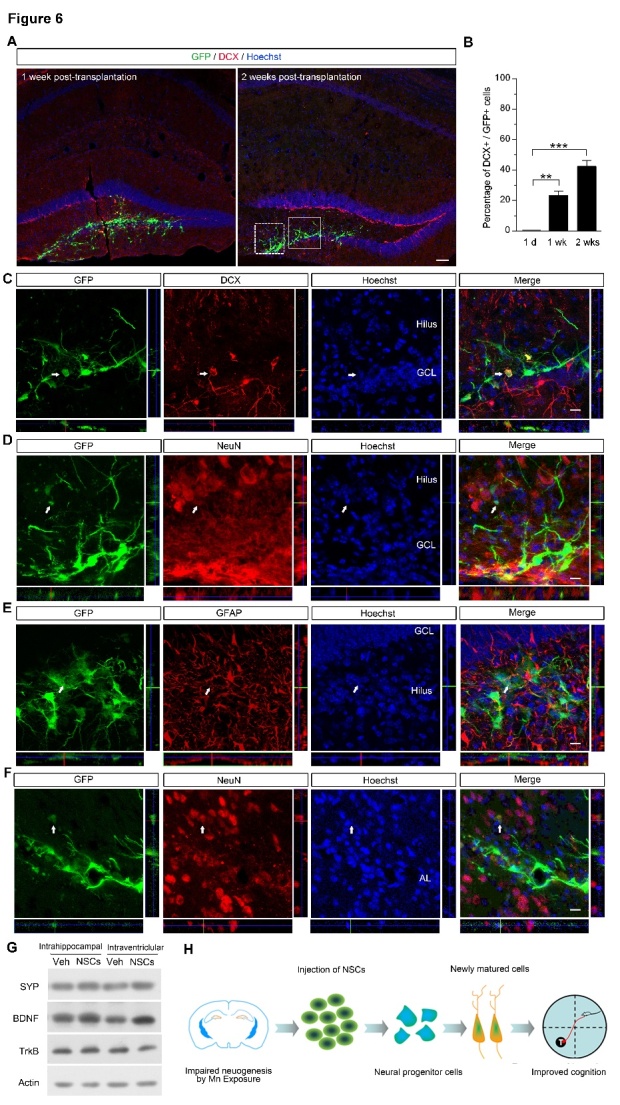
*In vivo* migration and differentiation of engrafted neural stem cells (NSCs) within the brains of manganese (Mn)-exposed mice. (A) Representative images showing that transplanted enhanced green fluorescent protein (eGFP)-labeled NSCs survived and partially differentiated into immature neurons in the hippocampal dentate gyrus (DG) of Mn-exposed mouse brains one (left) and two (right) weeks after transplantation. Double-cortin (DCX) was used as a protein marker for newly-generated neurons (red) and Hoechst staining was used to label DNA in cell nuclei (blue). (B) Quantitative analysis of the number of DCX-positive engrafted eGFP-positive cells one day, one week and two weeks after transplantation. *n* = 3; error bars represent means ± SEM; ** *P* <0.05 and *** *P* <0.01. (C) Magnified z-stack images of the solid line-boxed areas in A. The arrow points to a transplanted GFP cell with DCX immunoactivity. (D) Magnified z-stack images of dotted line-boxed areas in A. Some eGFP-positive (green) NSCs differentiate into NeuN-expressing neurons in the hippocampal DG two weeks after transplantation. (E) A small number of eGFP-positive cells (green) differentiate into astrocytes co-expressing glial fibrillary acidic protein (GFAP) (red) two weeks after transplantation. (F) Representative image showing the differentiation of engrafted NSCs into neurons in the AL two weeks after intracerebroventricular transplantation. An arrow indicates an eGFP-positive cell with immunolabeling for NeuN, a marker for mature neurons, that migrated from the original injection site. Z-stack images of the plane perpendicular to each field are shown in the right side and bottom of each image (C-F). Scale bars, 100 μm for A and 20 μm for C-F. DG, dentate gyrus; GCL, granule cell layer; AL, angulus lateralis. (G) Engrafted NSCs increase the protein levels of synaptophysin (SYP), brain-derived neurotrophic factor (BDNF), and tropomyosin receptor kinase B (TrkB) in hippocampal regions revealed by Western blotting. (H) Proposed schematic working model of NSC transplantation in restoring spatial cognition in Mn-exposed mice. Transplanted NSCs can survive, migrate, and differentiate in the host brain, eventually integrating into the local circuitries to repair the impaired neurogenesis induced by Mn exposure, resulting in improved learning and memory ability of Mn-exposed mice.

To explore the cellular changes underlying the restored cognitive performance induced by transplanted NSCs in the Mn-exposed mouse brain, we further assessed the migration and differentiation of the NSC transplants using immunofluorescent labeling. In groups that received intraventricular or intrahippocampal administration of cultured NSCs, we found that many transplanted NSCs were able to survive *in situ*, while some migrated far away from the injection sites. Much of these eGFP-positive cells differentiated and expressed DCX (Fig. 6A-C), a protein marker for newly-generated neurons, in the hippocampal DG of Mn-exposed mouse brains, with an increasing proportion expressing the marker over time after transplantation; the percentage of DCX+/eGFP+ engrafted cells was 23.267 ± 2.978 one week after transplantation and 42.467 ± 3.940 two weeks after transplantation (*P*= 0.0014 and *P*=0.0004, respectively, compared with one day post-transplantation) (Fig. 6B), indicating a tendency of the NSCs to integrate with the host tissue. Intriguingly, some of the transplanted cells were found to migrate into the granular cell layer of the DG; these cells had differentiated into functional neurons even two weeks post-transplantation, as they expressed the neuron-specific nuclear protein (NeuN) marker of mature neurons (Fig. 6D and F) or GFAP, an astrocytic glial cell marker (Fig. 6E). It is possible those cells integrated with the host tissue through elaborating axons and dendrites.

Given that NSC transplantation may have rescued the cognitive functional impairments induced by Mn exposure by increasing synaptic connectivity, we hypothesized that engrafted NSCs might establish new synaptic connections in the host brain. Thus, we directly examined the expression of some functional proteins key to cognitive function following NSC transplantation in Mn-exposed mouse brain using Western blotting. The expression levels of TrkB, BDNF, and SYP were upregulated within the hippocampal DG of Mn-exposed mice after NSC transplantation (Fig. 6G), suggesting that NSC-induced expression of these proteins may play a critical role in rescuing the cognitive deficits induced by Mn exposure. Taken together, these results imply that NSCs transplants likely improved cognitive function of Mn-exposed mice through enhancing endogenous neuronal connectivity within the host brain, and they support the potential efficacy of NSC transplantation to treat toxicity induced by Mn exposure (Fig. 6H).

## DISCUSSION

In this study, our finding that NSC transplantation improved the cognitive impairments in Mn-exposure mice is particularly exciting, as both intraventricular and intrahippocampal treatments significantly restored learning and memory in mice exhibiting impaired neurogenesis. More interestingly, IHC experiments further highlighted the ability of the transplanted NSCs to differentiate and integrate with the local circuitry, which may provide a mechanistic explanation for the functional recovery, though further investigation is required to elucidate the exact molecular mechanisms and to confirm the enhanced synaptic connectivity. These findings could ultimately lead to a systematic therapeutic strategy to treat heavy metal neurotoxicity.

Because of the wide use of Mn, research of Mn neurotoxicity still plays an important role in understanding heavy metal toxicology [4]. Recent studies have indicated that humans experiencing increased exposure to Mn exhibit poor cognitive performance, with particular impairments in attention, learning, and memory functions [4, 37]. Importantly, converging studies have found a significant correlation between Mn exposure and learning and behavioral deficits in children [15]. Consistently, *in vivo* animal models have also shown that Mn exposure results in motor and cognitive impairments, and limbic disorders [34] (Fig. 1). However, conventional pharmacological treatments for most neurotoxic conditions relieve some symptoms, but rarely alter the course of the disease or halt its progression. Current therapeutic approaches, including the use of metal complexes of salicylic acid are only palliative and they do not compensate for the massive and progressive cellular loss in the brains of patients with manganism [38]. Thus, one possible treatment strategy for Mn exposure would be to prevent and/or replace neuronal loss as the disease progresses.

Recent studies have suggested a potential role for NSCs in altering the etiology of Mn-related cognitive deficits [34]. NSCs have the potential for self-renewal and are multipotent cells that can generate functionally integrated cells in the nervous system of numerous mammalian species, including humans [32, 39]. It has, therefore, been suggested that NSCs are capable of compensating for lost or damaged cells in the injured brain, and NSC transplantation has been tested as a potential treatment for many types of neurological damage, including ischemia and traumatic brain injury, neurodegenerative disorders [40], as well as lead exposure [41]. Importantly, neurogenesis has been shown to be impaired in animal models of Mn exposure [30] ; it slows the proliferation of NSCs *in vivo* and *in vitro* [42, 43]. The hippocampal region of the developing brain is especially vulnerable to Mn-induced neurotoxicity and the subsequent spatial learning and cognitive impairments [34]; this, coupled with the recent observations that impaired neurogenesis in animals exposed to Mn during development closely resembles the structural disruptions common to some neurodegenerative diseases [4], prompted us to investigate if these functional deficits could be restored by NSC transplantation in young Mn-exposed mice.

Over the past two decades, NSC-based therapies have grown to represent a promising treatment strategy for many brain disorders [23]. Multiple studies using NSCs have provided proof of concept, demonstrating benefits to brain function after CNS injury [40]. Our findings indicate that exogenous NSC transplantation restored the spatial learning deficits in Mn-exposed rats to a significant extent. Although morphological deficits improved differently between the two methods of intracerebral injection, the significant restoration of cognitive function was found weeks after transplantation. Our results directly showed that following intracere-broventricular injection, exogenous NSCs can cross the blood-brain barrier to arrive at the hippocampal DG where they can differentiate into mature neurons (Fig. 3 and 6), providing further evidence of the functional recovery induced by these cells. In addition, direct injection of NSCs into the hippocampal parenchyma also resulted in sufficient engraftment, and importantly, the transplanted NSCs rapidly migrated away from the injection sites along known neural stem cell migratory pathways [42, 44]. Small numbers of these cells further differentiated into neuronal or glial cell types, with similar results seen following intraventricular injection (Fig. 6). These findings are consistent with previous studies in other kinds of animal models that have shown that NSCs can be transplanted into the brain, that they can migrate throughout the CNS, and can survive for several months [40]. Our study provides direct *in vivo* evidence that transplanted NSCs can differentiate into mature cells and supports the notion that these newly generated cells play an important role in hippocampal function.

This NSC-based therapeutic approach can improve brain function via several alternative mechanisms that, alone or in combination, could include compensating for damaged cells, providing neuroprotection through neurotrophin secretion, and by modulating inflammation. For instance, reinnervation of components in the host brain through a graft and/or a more complete integration of the transplanted cells into the host circuitry with the establishment of reciprocal graft-host connections has been proposed to markedly induce functional recovery [40]. The effective integration of transplanted neurons with the host neurons likely improves cognition by enhancing endogenous neuronal connectivity [45]. In the present study, the beneficial effects of NSC transplantation on cognition might have been mediated by the extent to which the graft was able to become sufficiently integrated several weeks post-transplantation; evidence of this transplanted cell integration was observed by double-labeling IHC in brain sections (Fig. 6). We found that many of the exogenous NSCs could survive, migrate, and differentiate successfully within the DG of the host hippocampus, and that this integration was associated with significant improvements in cognitive function in the behavioral tests. These transplanted NSCs differentiated into neurons and astrocytes, leading to enhanced neurogenesis and improved cognitive ability.

Another possible explanation for the improved neurobehavioral deficits could be linked to trophic support provided by the transplanted cells [46]. NSC-derived cells may release neurotrophins or neuro-transmitters that can diffuse throughout the hippocampal area, facilitating the recovery of the damaged endogenous neuronal circuitry [47]. Alternatively, the transplanted cells could preserve neuronal function by providing beneficial enzymes or by modulating inflammatory processes [48], though whether transplantation of NSCs can improve the microenvironment after Mn exposure by regulating neurotransmitter release needs to be further elucidated. Previous studies have shown that transplantation of bone marrow-derived cells may provide neuroprotection against strokes by reducing inflammatory responses or by secreting certain neurotrophins or promoting their release [27]. Engrafted NSCs can counteract oxidative stress and inhibit the inflammatory responses associated with disease states by upregulating antioxidant enzymes [49]. Consistent with these observations, our experimental results showed that the transplantation of NSCs in the brain can promote the secretion of BDNF, suggesting NSCs can modulate inflammatory responses through neurotrophic factors (Fig. 6G). Taken together, our results showed that grafted NSCs were able to ameliorate the neurobehavioral deficits induced by Mn exposure via one or more of the above mechanisms, thereby facilitating the repair of the perturbed neuronal cytoarchitecture of the hippocampus, which is essential for optimal memory formation and spatial cognitive functions [50]. To our knowledge, this is the first report demonstrating the therapeutic efficacy of intracerebral NSC transplantation in an animal model of Mn exposure.

While the transplantation results we present here are encouraging, particularly given the fact that only a limited number of NSCs were injected and an even smaller number of those survived to undergo migration and proliferation in the hippocampus (Fig. 6), the functional recovery was only partial (Fig. 5). To further improve on the results reported here and to ensure that a greater number of NSCs survive and proliferate in the brain following transplantation, the donor NSCs could be modified to express certain neurotrophins to enhance the cognitive recovery of Mn-exposed mice. Currently, the transfection efficiency of retroviral vectors remains low based on the current protocols of neurosphere culturing [51]. This combined with the high fluorescent background signals in the host brain [40] makes it difficult to provide conclusive results. Thus, for these initial studies we chose to use this crude but direct method of NSC preparation in order to avoid possible immune responses elicited by foreign antigens from the damaged cells in the host brain.

In conclusion, we showed that the transplantation of *in vitro* cultured NSCs directly into the brains of Mn-exposed mice can elicit significant improvements in cognitive behaviors. This is the first report of this approach for this disease, and we believe there may be potential clinical application for treating patients with severe toxicity from Mn exposure. While similar intracerebral transplantation studies have been carried out in other neurotoxic disease models such as lead exposure using cultured fibroblasts or NSCs [41], the fact that NSCs can be easily obtained and have excellent migratory capacity in the CNS makes them prime candidates for future therapeutic evaluation. However, the optimal conditions for minimizing immunological rejection and ensuring the long-term survival and synaptic integration of these cells in the human CNS remain to be determined. Although NSCs has been applied to treat patients with many neurodegenerative diseases, a multicenter, double-blind, randomized, placebo-controlled study has yet to be carried out to evaluate the overall efficacy of using NSCs to treat toxicity from Mn exposure. Furthermore, whether NSCs should be prescribed alone or in combination with other therapeutic strategies should be assessed in future clinical trials. Nevertheless, future efforts will be directed toward improving on the results reported here to achieve better therapeutic effects. Taken together, our findings indicate the potent effect of transplanted NSCs for improving brain function following heavy metal exposure and suggest that the further development of NSC-based therapies could provide a viable approach to treat severe Mn exposure and other related diseases.
